# Thoughts on the NHS

**Published:** 1991-09

**Authors:** M. D. Read

**Affiliations:** Consultant Obstetrician and Gynaecologist Gloucester Royal Hospital


### Thoughts on the NHS

**M. D. Read, MD, FRCS Ed, MRCOG, FRACOG**
Consultant Obstetrician and Gynaecologist
Gloucester Royal Hospital

### DO REDUCTIONS IN CLINICAL SERVICE SAVE MONEY?

It is common practice for management when faced with financial overspends to combat this by reducing services and closing wards or departments. But does this really save any money? It is a paradox of the present National Health Service that hard working units fall into an efficiency trap. The more work that is done, the more patients who are seen and treated, the more consumables are used and more money is spent. Any reduction in this practice means the waiting list will increase, which is contrary to the fundamental beliefs, or so we are told, of the Department of Health. Faced with the fact that 75% of any budget is pay, add to that the overheads of the premises and their maintenance, there is but a small fraction of the budget left to represent the cost of treating patients. Thus to make any significant saving in the overall budget, one has to make draconian cuts in the service. Indeed, because the absolute costs to treat each patient are small, one may as well continue working. The assertions of the Government that this sort of problem will be over-come with the brave new world of money following patients, ignores the fact that one needs the resources **before one can treat the patients** not after. Let us look more closely at some of the more common practices adopted in saving money.

**Closure of outpatient departments for limited times.**

This is common practice around public holidays, especially at Christmas time. The figures put forward by management suggesting massive savings are a complete myth as the figures appear to be simply the overall cost of running the department divided by the time for which it is closed. This takes no cognisance of the facts that:

1. The buildings are still present with their overheads;
2. The staff have to be paid, they cannot be laid off without pay for short terms;
3. The patients who are not seen in the weeks of closure will be seen as a catchup phenomenon after. Thus within the financial year, the patients will still be seen and all the costs associated with them including laboratory investigations, X-rays, etc., will still be generated. If there were no catchup phenomenon it would simply transfer so many more patients onto the following year's waiting list.

Thus, there is no validity in the argument that closing a department saves money. Certainly around Christmas and the New Year it is found that many patients do not keep their appointments. It may well be that a number of staff would be quite happy to take leave at that time of year and thus it is a convenient time to close the department, but let us at least be honest in our motives for closure.

**Closure of beds**

The only bed closures which are going to generate any savings are when whole wards can be closed and the staff compliment reduced.

Certainly if 7 day wards can be converted to 5 day wards this can generate considerable savings, as paying staff at weekends is disproportionately expensive. Also, a greater use of day case facilities where the wards are not required at night will save money but simply not using a few beds within a ward that is still open saves nothing.

**Cutting out overtime for ancilliary staff**

Overtime for all members of hospital staff except medical staff is more expensive than basic salary thus, in isolation, one can see that by reducing overtime payments, money might be saved. However, let us look at the overall effect of this action. Recently, in one hospital overtime for the portering staff was stopped. The result of this meant re-allocation of the portering staff during the working day and there was an associated reduction in the provision of porters to fetch patients from the ward to the operating theatre. As a result, less work was put through theatre and if one looked in the sitting room one could see Surgeons, Anaesthetists, Theatre Nurses and Operating Department Assistants and Orderlies who could not work because there was no patient available. At 2.30 on a Friday afternoon, one could count over £200,000 per annum salary sitting in the room, waiting for patients for the sake of £5 per hour!!! Almost all hospitals face the financial dilemma. It is an uncomfortable fact that the ever-increasing demand for treatment, the increasing expectations of the public which is infinite cannot be matched by finite resources, however much is provided. Management often finds itself in a Catch 22 situation, as any attempts to save money by closures increase the waiting list which is a declared target of the Government. How then can the dilemma be resolved? The only thing I am certain of is the answers will not be found in this letter! It is certainly not closures and whatever solution is found it is going to be unpopular politically and we may have to wait a long time for someone to grasp the nettle. Sooner or later some form of rationing by type of treatment is going to be necessary rather than simply rationing by waiting list, which takes no account of the importance of the procedure to the individual or to the nation at large.

**WAITING LISTS**

What is a waiting list? A question which has exercised the minds of philosophers almost as much as the meaning of life itself. A waiting list is a perception. Ministers perceive it as an election issue and vote loser. The Department of Health perceives it as inefficiency at best and a contrived plan to boost private earnings at worst. General Managers perceive it as a financial issue now there is performance related pay. To the Clinician a waiting list is a reflection of inadequate resources. There is no doubt that each of the interested parties can provide examples to illustrate their point. From the Department's point of view we can look at Merseyside where the number of patients waiting more than 2 years for surgery was reduced from 4,000 to 17. Now, we are not told how this was achieved, but is reasonable to assume that a significant number of people either moved out of the area, no longer wished to have their operation, had had the procedure performed elsewhere, or had died. On top of that there may be a reduction because of better throughput with more efficient use of resources. That such a magnitude of improvement could be achieved does suggest the Department has a point. However, if one looks to the staffing levels in the South West region, compared with the national average, one can readily see that the Clinician's viewpoint has considerable merit. For most regions and districts, of course, the truth, if such a thing can ever be described, is somewhere inbetween the two extremes of the spectrum.

Any attempt to solve waiting list problems by applying a "blanket remedy" is doomed to failure when what is really required is an individual appraisal and response to the needs of each district and indeed each speciality.

Every year waiting list initiative monies are allocated. Every year we ask that these be allocated on a recurrent basis so that we can actually plan how best to use these resources. We are told that it is a non-recurrent item and that we cannot guarantee them every year. Thus the notorious waiting list initiative crumbs are thrown yearly. The result is wasteful with only a temporary drop in the waiting list in one or two areas. There has never been shown to be sustained decrease in the waiting list. It is at best simply a temporary expedient and these resources could be put to much greater use if they were available on a recurrent basis. There are two ways to use this waiting list initiative money effeciently. The first is to employ people on a sessional basis so that you can make inroads into a waiting list, if indeed it is shortage of personnel and operating time that is the cause of the wait rather than the shortage of beds.

The second way is actually much simpler and does not require one to set up anything special. One simply makes a list of the top 10 operations on the waiting list, see Table 1. One then finds out the contract price for this procedure from the adjacent Health Districts (preferrably self-governing trust) and use the waiting list initiative money to block purchase a number of these operations. By doing this each year one can in turn attack these 10 top operations and as number 1 gets down to politically acceptable levels, one will move on to the 2nd and so on, or one may creatively divide the money to reduce several lists at once (Table 2). The same end being achieved with no hassles!

Table 1 **TOP 10**

| CASE | NO | UNIT COST* | TOTAL COST** |
|---|---|---|---|
| Cataract Extraction/ Lens implant | 686 (511) | 785 | 538,500 |
| Dental Extraction/ Clearance | 595 | 33 | 19,600 |
| Varicose Veins | 405 | I/P 640 | 259,200 |
| | | D/C 364 | 147,400 |
| Cystoscopy | 292 | I/P 610 | 278,100 |
| | | D/C 120 | 35,040 |
| Hernia | 251 | I/P 880 | 221,000 |
| | | D/C 424 | 106,400 |
| Prostate | 237 | 1,295 | 307,000 |
| Hip Replacement | 205 (125) | 3,200 | 656,000 |
| Tonsils/Adenoids | 189 | 634 | 119,800 |
| SMR/Septo rhinoplasty | 189 | 761 | 143,800 |
| Knee Replacement | 121 (111) | 3,585 | 433,800 |

* Rounded to nearest £1
**Rounded to nearest £100
( ) Number of cases which could be bought for £400,000
I/P In Patient
D/C Day Case

Table 2

For Waiting List Initiative money of £400,000 one could:—
a) reduce cataract list by 75%
or b) reduce hip replacement list by 60%
or c) reduce knee replacement list by 92%
or d) clear all : T & A
SMR
Dental extraction
Hernias (assuming day cases)
or e) clear all : Varicose veins
Cystoscopies
Hernias
Dental extraction
30 hips
(Assuming day cases)
or f) clear all : Dental extractions
T & A
SMR
Cystoscopies (assuming day case)
320 day case hernias
or g) clear prostate list and hernia lists (assuming day cases).

### THE OUTPATIENT CLINIC

In the drive to make Outpatient attendances more personal and less like the cattle markets of old, there now has been a dramatic change, each patient now having individual times rather than the old block booking system. I well remember, not all that many years ago, as a senior registrar working in a busy unit in the North West of the country, where the cattle market system ruled supreme, patients came up, were given a numbered disc and were seen in the order of the numbers. It was commonplace for ladies to come and collect a number and then go off into town to do their shopping. They would come back a couple of hours later still in plenty of time for their turn! Whilst the results of recent changes are in general positive, there are a few adverse effects which seem to go unnoticed.

Imagine the patient living some distance from the hospital who has spent more than an hour travelling on the bus or train to get to the hospital and is looking forward to a rest and a cup of tea. Having brought a book or magazine with them and looking forward to a chat with the other patients in the waiting room to alleviate their anxiety. They feel very ill at ease indeed when they find they are the only people in the waiting room, feeling rather exposed without protection of anonymity. They feel almost cheated when they are back out on their way home within 30 minutes of arriving, having been seen!

What of problems from the staff's point of view? What happens when a patient turns up late? Then we have a dilemma. Do we see the patients in appointment order so that those with later appointments have to wait because of the late arrival, or do we see the patients at the appointment time, thus the patient who is late has to wait until the end of the clinic? Within individual time slots all we need is one late patient or a defaulter and we have doctors waiting around for patients, an eerie experience indeed.

In addition, there is the paradox in these times of market economics in the Health Service. If there is no-one in the waiting room there is no-one to use the refreshment facilities and income generation fails.

